# Piroxicam and paracetamol in the prevention of early recurrent pain and emergency department readmission after renal colic: Randomized placebo‐controlled trial

**DOI:** 10.1111/acem.14996

**Published:** 2024-08-19

**Authors:** Rahma Jaballah, Marwa Toumia, Rym Youssef, Khaoula Bel Haj Ali, Arij Bakir, Sarra Sassi, Hajer Yaakoubi, Cyrine Kouraichi, Randa Dhaoui, Adel Sekma, Asma Zorgati, Kaouthar Beltaief, Zied Mezgar, Mariem Khrouf, Wahid Bouida, Mohamed Habib Grissa, Jamel Saad, Hamdi Boubaker, Riadh Boukef, Mohamed Amine Msolli, Semir Nouira

**Affiliations:** ^1^ Research Laboratory LR12SP18 Monastir University Monastir Tunisia; ^2^ Emergency Department Sahloul University Hospital Sousse Tunisia; ^3^ Emergency Department Fattouma Bourguiba University Hospital Monastir Tunisia; ^4^ Emergency Department Hached University Hospital Sousse Tunisia; ^5^ Department of Imaging and Interventional Radiology Fattouma Bourguiba University Hospital Monastir Tunisia

**Keywords:** nonsteroidal anti‐inflammatory drugs, paracetamol, readmission, recurrent pain, renal colic

## Abstract

**Objective:**

Renal colic (RC) is a common urologic emergency often leading to significant pain and recurrent hospital visits. This study aimed to compare the efficacy and safety of piroxicam versus paracetamol in preventing pain recurrence and hospital readmission in patients treated for RC and discharged from the emergency department (ED).

**Methods:**

A prospective, randomized, single‐blind trial was conducted in four EDs. Eligible adults with RC were randomized to receive oral piroxicam, paracetamol, or placebo for 5 days post–ED discharge. Primary outcomes included pain recurrence and ED readmission within 7 days. Secondary outcomes included time to recurrence and treatment‐related side effects.

**Results:**

Of 1383 enrolled patients, no significant differences were observed among the groups regarding baseline characteristics. Pain recurrence rates within 7 days were 29% (95% confidence interval [CI] 24.9%–33.2%) for piroxicam, 30.3% (95% CI 26.1%–34.5%) for paracetamol, and 30.8% (95% CI 26.6%–35.0%) for placebo, with no significant between‐group differences (*p* = 0.84). Among patients experiencing recurrence, the majority encounter it within the initial 2 days following their discharge (86% in the piroxicam group, 84.1% in the paracetamol group, and 86% in the placebo group, respectively). ED readmission rates were similar across groups: 20.8% (95% CI 17.1%–24.5%) in the piroxicam group, 23.8% (95% CI 19.9%–27.7%) in the paracetamol group, and 22.9% (95% CI 19.1%–26.8%) in the placebo group (*p* = 0.52). The piroxicam group reported significantly higher adverse effects compared to others.

**Conclusions:**

Piroxicam and paracetamol did not demonstrate efficacy in preventing pain recurrence or ED readmission within the first week following RC treatment.

## INTRODUCTION

Renal colic (RC), characterized by its excruciating pain, is a prevalent urologic emergency caused by urolithiasis, and a leading reason to seek immediate care in emergency departments (EDs).^1^ With an escalating incidence estimated at 14% in England and 10.1% in the United States, this condition exerts a substantial impact on quality of life^2^, ^3^ and underscores the concern for the imperative need for effective management strategies. Most patients with RC are managed as outpatients, which highlights the importance of post‐ED discharge analgesic treatment to prevent recurrence and ensure patient well‐being.^1^ Importantly, recurrence rates within the first month after hospital discharge could be as high as 10%, accentuating the challenge in mitigating this major event.^4^, ^5^ Additionally, persistent pain required frequent ED visits and rescue reinterventions, perpetuating a cycle of discomfort and health care resource utilization.^6^ Despite attempts to implement preventive measures like dietary and drug interventions, their efficacy remains limited.^7^, ^8^, ^9^, ^10^, ^11^ Germane to the ED, there is currently a lack of comprehensive studies and recommendations addressing the post–RC treatment to prevent the early recurrence. In routine clinical practice, patients are commonly discharged with nonsteroidal anti‐inflammatory drugs (NSAIDs) despite the lack of conclusive evidence.^12^ Indeed, studies investigating the effectiveness of NSAIDs as preventive therapy or alternative treatments remain notably scarce. Our study aim is to compare the benefit of oral NSAIDs and paracetamol for patients discharged from the ED following treatment of RC, with a specific emphasis on recurrence of pain and need for hospital readmission.

## PATIENTS AND METHODS

### Study design

Over the course of 5 years, from January 2016 to December 2021, a prospective, randomized, single‐blind study was carried out in four Tunisians EDs (Fattouma Bourguiba University Hospital, Sahloul University Hospital, Farhat Hached University Hospital, and Jammel Regional Hospital). The research protocol received approval from the ethics committee of our institution. Written informed consent was obtained from all eligible participants, and the study adhered to the reporting guidelines outlined in the Consolidated Standards of Reporting Trials (CONSORT). The study was registered in clinical trials (NCT02304783).

### Participants

Patients considered for inclusion were adults aged 18 and older who presented to the ED with a chief complaint of abdominal or flank pain and a final diagnosis of RC and were discharged with a numeric rating scale (NRS) < 3. The diagnosis of RC was based on history and clinical findings with presence of hematuria in urinary test or urinary tract stone identified by ultrasonography or radiologic imaging, including CT scan. Patients were instructed to avoid the use of over‐the‐counter (OTC) analgesics during the study period to ensure accurate assessment of the trial medications' effectiveness. Patient screening and inclusion were not interrupted during off days.

### Pain assessment

Pain on the NRS was assessed by trained clinical research associates (CRAs) who previously underwent a comprehensive training program to ensure consistency and accuracy in pain assessment. The training included detailed instructions on the use of the NRS and supervised practice sessions. Additionally, periodic refresher training sessions were conducted to maintain the high quality of assessments throughout the study. We excluded patients with documented or suspected pregnancy, breastfeeding, allergy or contraindications to NSAIDs or paracetamol, known renal or hepatic dysfunction, unmanaged diabetes, history of peptic ulcer disease or gastrointestinal hemorrhage, history of cardiac arrhythmia, severe coronary artery disease with anticoagulant medication or coagulation disorders, and complicated RC (infection, uncontrolled pain, anuria) or in case of need for hospital admission.

### Baseline data collection

Patients were approached and enrolled by the CRA after the decision to discharge was made by the attending physician. Patients were briefed on the study's objectives, with an emphasis on data confidentiality and anonymity. Data regarding sociodemographic characteristics, clinical history, and standard laboratory tests were collected using validated case report forms. Baseline examinations encompassed a clinical examination, kidney urinary bladder radiograph, cytobacteriological urinary analysis, renal ultrasound, creatinine measurements, and NRS at discharge. To ensure a high follow‐up rate, we implemented several strategies including comprehensive contact information using multiple phone numbers. In addition, to facilitate reliable communication, a dedicated team of CRAs was assigned to conduct follow‐up calls and visits when required to maintain consistent and regular contact with participants.

### Randomization and blinding

Participants were randomly assigned to one of three groups: patients in the first group received an envelope containing one pill of piroxicam (20 mg) orally and one pill of placebo, every day for 5 days (piroxicam group). Patients in the second group received 2 g oral paracetamol per day (1 g at the morning and 1 g in the evening) for 5 days (paracetamol group). The third group received one pill of placebo in the morning and one pill in the evening for 5 days (placebo group). Participants were administered only the study medications (piroxicam, paracetamol, or placebo). No additional treatments or medications were provided by their treating physicians during the study period. Patients were instructed to avoid the use of OTC analgesics during the study period. Randomization was accomplished using a computer‐based random sequence generator. The allocation sequence was kept by a CRA not involved in enrollment, treatment, or assessment. Group assignments were concealed from the investigators, providers, and participants involved in the study. Statistical analysis was performed by a blinded investigator. Allocation was revealed after conclusion of the study.

### Follow‐up

Patients were contacted via telephone 7 days following ED discharge. Patients were asked if they experienced RC symptoms (with at least an intensity > 3 at a NRS ranging from a minimum of 0 to a maximum of 10), ED readmissions, and side effects related to protocol treatment.

### Outcome criteria

Primary outcome included recurrence of RC and ED readmission within 7‐day follow‐up. Secondary outcomes included mean time to recurrence of pain and occurrence of side effects.

### Statistical analysis

Based on event rate (pain recurrence and readmission) at 7 days estimated at 30% and using an alpha value of 0.05, a study of 1038 patients will have 95% power to detect a 5% decrease in absolute risk in the treatment group compared with the placebo group. The sample size was increased by 30% to compensate for the number of patients randomized but lost to follow‐up, for a total sample size of 1350 patients. Analysis was undertaken on an intention‐to‐treat basis. Patients were removed from analysis after randomization only if recruitment was an unequivocal protocol violation (i.e., no consent had been recorded or if they had previously been recruited) or if the patient withdrew from the trial prior to any treatments having been administered. The study was designed to test the superiority of one of the active protocol treatments over placebo. Nonparametric statistical techniques were used for the continuous data, as these data were not normally distributed. Patient characteristics and outcome measures were reported as means with standard deviations (SDs) or medians and 95% confidence intervals (CIs), as appropriate. Descriptive and inferential statistical analyses (Kruskal–Wallis, Mann–Whitney rank sum, or Friedman tests for continuous variables; Fisher exact or chi‐square tests for categorical data) were performed as appropriate. Pairwise comparisons were used in our analysis with Bonferroni adjustment. All tests were run as two‐tailed and deemed significant if <0.05. The statistical analysis was carried out using the SPSS software 2.0.

## RESULTS

**FIGURE 1:**
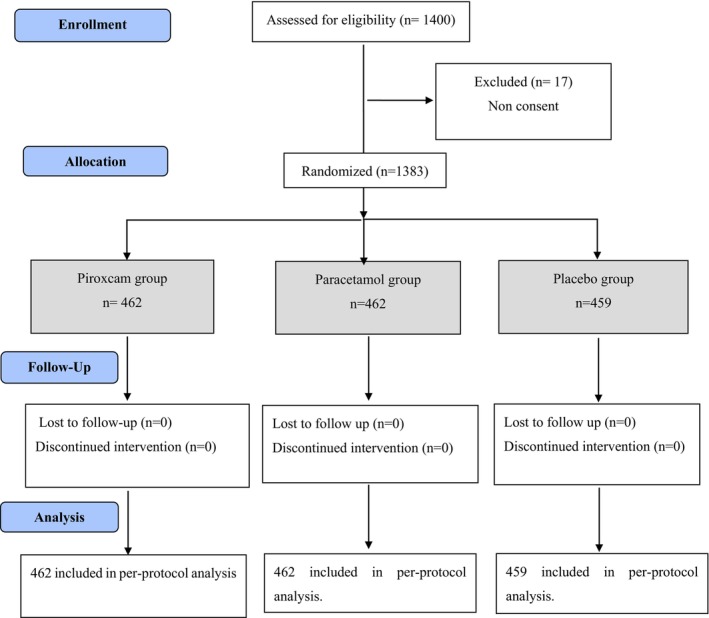
Trial profile.

**FIGURE 2:**
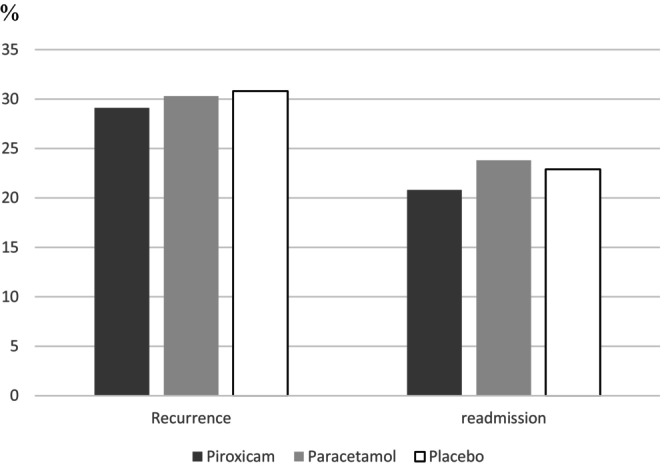
Recurrence and ED readmission rate in the protocol groups. There is no difference between the three groups of study.

**FIGURE 3:**
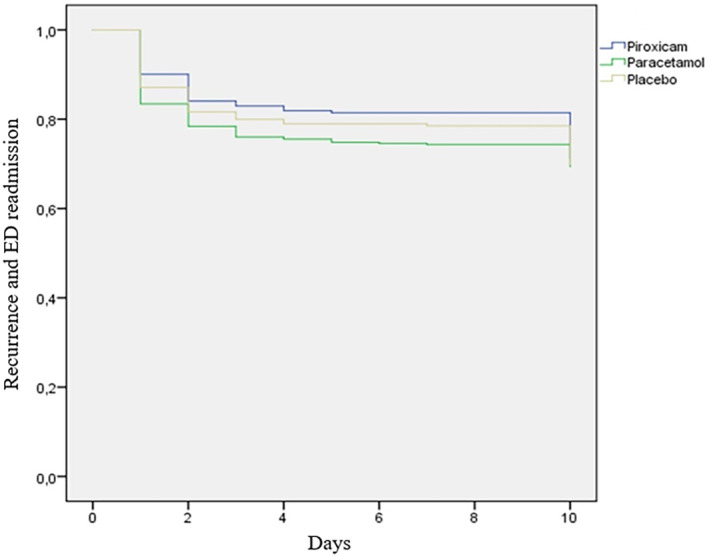
Kaplan–Meier curve showing the probability of primary outcome event over time (recurrence and ED readmission) in the three study groups. There is no difference between the three study groups (log‐rank 0.9 *p* = 0.2).

A study enrollment flow diagram is displayed in Figure 1. A total of 1400 patients were eligible; 17 patients withdrew consent before randomization. A total of 1383 patients ultimately received the study medications, 462 in the piroxicam group, 462 in the paracetamol group, and 459 in the placebo group (Figure 1). The three study groups demonstrated comparability regarding baseline demographic and clinical characteristics. The mean ± SD ages were 42.9 ± 12 years for the piroxicam group, 43.6 ± 13 years for the paracetamol group, and 43.5 ± 13 years for the placebo group (*p* = 0.92). The previous medical history of nephrolithiasis was similar in all three groups, with no statistically significant differences observed: 34.5% in the piroxicam group, 33% in the paracetamol group, and 32.4% in the placebo group (*p* = 0.81). Among the 1383 study participants, 364 had a history of RC, 128 (27.7%) in the piroxicam group, 123 (26.6%) in the paracetamol group, and 113 (24.6%) in the placebo group (*p* = 0.55). Pain levels at ED discharge were comparable among the groups (*p* = 0.86) (Table 1). More than half (55%) of the patients (*n* = 761) were investigated with ultrasonography showing a urinary tract stone in 160 patients (21%) and a pyelocaliceal dilation in 282 patients (37%). The overall prevalence of recurrence across all three groups reached 30%. After discharge, pain recurrence was reported by 134 patients in the piroxicam group (29%, 95% CI 24.9%–33.2%), 140 in the paracetamol group (30.3%, 95% CI 26.1%–34.5%), and 141 in the placebo group (30.8%, 95% CI 26.6%–35.0%). No significant differences observed between the groups (*p* = 0.84; Figure 2). Among patients experiencing recurrence, the majority encounter it within the initial 2 days following their discharge from the ED. Specifically, within this time frame, recurrence rates were observed at 86% in the piroxicam group, 84.1% in the paracetamol group, and 86% in the placebo group (Figure 3). Overall, 311 (22.4%) patients included were readmitted to the ED, 96 (20.8%, 95% CI 17.1%–24.5%) in the piroxicam group, 110 (23.8%, 95% CI 19.9%–27.7%) in the paracetamol group, and 105 (22.9%, 95% CI 19.1%–26.8%) in the placebo group (*p* = 0.52). The mean time to readmission was 2 days (IQR 1–3 days) for the piroxicam group, 1 day (IQR 1‐2 days) in the paracetamol group, and 1 day (IQR 1–2 days) in the placebo group (*p* = 0.99; Figure 2). Thirteen patients (11 in the piroxicam group, one in the paracetamol group, and 1 in the placebo group; *p* < 0.001) experienced side effects, including epigastric pain and vomiting. Additionally, four patients presented with minimal allergic reactions including pruritus and rash, with two in each the piroxicam and placebo groups. No severe side effects were recorded throughout the study.

**TABLE 1:** Baseline population characteristics.

	Piroxicam	Paracetamol	Placebo
Age (years)	42.5 (41.8–44.2)	43 (42.4–44.9)	43.6 (42.3–44.7)
Male gender	262 (56.2)	222 (53.2)	229 (54.5)
Previous medical history			
RC	128 (27.7)	123 (26.6)	113 (24.6)
Nephrolithiasis	184 (34.5)	176 (33.0)	173 (32.4)
Urinary tract infection	39 (8.4)	30 (7.2)	44 (10.5)
Clinical signs			
Temperature (°C)	37 (36.8–36.9)	36.8 (36.7–36.9)	36.8 (36.6–36.8)
Heart rate (beats/min)	86.3 (84.7–87.8)	86 (83.9–86.7)	85.3 (84.6–87.9)
Systolic blood pressure (mm Hg)	126 (125.2–128.2)	126 (124.9–128.0)	126 (125.6–128.6)
Diastolic blood pressure (mm Hg)	73 (72.5–74.5)	73 (71.4–73.5)	72.4 (70.9–73.1)
Imaging findings	255 (55.1)	254 (55.0)	252 (54.9)
Stone	54 (21.2)	51 (20.2)	55 (21.7)
Pyelocaliceal dilatation	100 (39.0)	100 (37.3)	96 (38.0)

*Note:* Data are reported as median (95% CI) or *n* (%).

Abbreviation: RC, renal colic.

## DISCUSSION

In this randomized, double‐blind, placebo‐controlled clinical trial, we found no difference between piroxicam, paracetamol, and placebo for the prevention of RC recurrence and hospital readmission within the first week after ED discharge. In piroxicam group, significant higher adverse effects were reported; no major side effects were noted during the study.

Despite the high frequency of RC and a substantial percentage of pain recurrence following discharge from the ED, studies addressing preventive strategies are very limited. The poor management of RC cases after ED discharge has a significant impact on quality of life, leading to prolonged discomfort and potential complications. Grenabo and Holmlund^13^ in 1984 performed, to our knowledge, the first randomized placebo‐controlled trial to evaluate the prophylactic effect of indomethacin on RC recurrence in 78 patients. They concluded that indomethacin administrated for 7 days reduced the frequency of severe pain and reduced the pain‐free interval without influencing the stone passage. One year later, Kapoor et al.^14^ conducted another randomized double‐blind placebo‐controlled trial to determine the effects of indomethacin suppositories in the relief of RC and prevention of its recurrence. Their findings added another support to the use of indomethacin in the prevention of recurrent pain secondary to ureteral calculi. In the study by Laerum et al.,^15^ 80 patients were divided in two groups, 41 received diclofenac for 7 days and 39 received matching placebo tablets. They found that diclofenac is effective for reducing recurrence of new colic episodes with a significant reduction of hospital readmission. Specifically, the readmission rate to an Oslo emergency hospital or other hospitals was 10% in the diclofenac group, significantly lower than the 67% observed in the placebo group (*p* < 0.001). However, the stone passage rate appears not to be affected. In these three studies reporting the benefit of NSAID ins RC, the combined sample size was much lower than the number of patients included in our current study. This might partly explain the difference between our study and previous studies’ results. The ED revisit rate in our study is notably higher than previously reported rates. High nephrolithiasis prevalence^16^with effect of heat and humidity^17^ are conditions that could exacerbate symptoms and lead to more ED visits. We can also speculate that the dose of paracetamol used in this study, along with the twice‐daily dosing frequency, were relatively lower than what was allowed (up to 4 g) which may contribute to its suboptimal effectiveness. Furthermore, the local inflammatory response and heightened pressure in the urinary tract, key contributors to RC pain, may not be adequately mitigated by limited target mechanism analgesic.^18^ This underscores the importance of exploring alternative multimodal therapies that specifically target several pain mechanisms to counteract the multiple pathophysiological dimensions of RC. Additional studies are required to examine the efficacy of these therapeutic options combining opioid and nonopioid analgesics and eventually medical expulsive therapy.^19^ Early surgical intervention may also be considered to decrease the prevalence of RC recurrence.

## STRENGTHS AND LIMITATIONS

The strength of our study is that it is the largest randomized placebo‐controlled trial focusing on the prevention of early pain recurrence and hospital readmission after treatment of RC; the study reached full power, thereby minimizing chances for a Type I or Type II error. However, this study has several limitations that deserve mention. First, the choice of piroxicam may be important in the interpretation of the results as other analgesic NSAIDs could be used for comparison. This should be further investigated; however, available data showed that piroxicam is effective in RC.^20^, ^21^ Second, in this study we did not practice diagnostic imaging for all patients with suspected urolithiasis, which means that data on the number, location, and size of urinary stones were lacking. These factors may affect pain recurrence and lead to differences in outcomes.^22^, ^23^ Third, spontaneous auto administration of rescue analgesia by some of our patients is possible. This information was not available, but this could influence the primary end point in our study. Additionally, the concept of preventive analgesia was not explicitly addressed, warranting further investigation into its potential impact on RC recurrence. Fourth, a notable limitation of our study is the absence of objective data on patient adherence to the treatment regimens. We did not collect specific adherence metrics such as the median number of leftover pills in each group. However, we took steps to encourage adherence through regular follow‐up phone calls, where RCA reminded patients of the importance of taking their medications as prescribed and encouraged them to report any challenges they faced. Future studies should incorporate methods such as pill counts or electronic monitoring to provide a more accurate assessment of adherence. Lastly, it is important to clarify that these results do not apply to as‐needed analgesics for pain, rather that we studied the prophylactic analgesia. This means that emergency physicians should still prescribe appropriate analgesics upon discharge for patients to manage pain when needed.

## CONCLUSIONS

The findings of this randomized controlled trial suggest that nonsteroidal anti‐inflammatory drugs and paracetamol were not useful to prevent pain recurrence and hospital readmission within the week after renal colic. Further trials should address the effectiveness of other treatment strategies including multimodal analgesic approach.

## CONFLICT OF INTEREST STATEMENT

The authors declare no conflicts of interest.

## Supporting information


Data S1.
