# Supplementary material for: Piroxicam and paracetamol in the prevention of early recurrent pain and emergency department readmission after renal colic: Randomized placebo‐controlled trial
Source: Acad Emerg Med. 2024 Aug 19;32(2):158–64. doi: 10.1111/acem.14996 (PMC11815999; doi:10.1111/acem.14996)
Supplement: Supplementary file 1 — Data S1. [file ACEM-32-158-s001.docx]

**Paracétamol versus piroxicam versus placebo dans le traitement de sortie des Urgences des coliques néphrétiques**

N° Fiche : _________________________

Date : ______ / ______ / ____________ Nom : ____________________ Prénom : __________________

Age : ____________ Sexe : M □ F □

Adresse : ______________________________________________ N° tel : _____________________

| ***Critères d’inclusion*** | oui | non |
| --- | --- | --- |
| Age > 18 ans |  |  |
| Consentement à la participation à l’étude |  |  |
| Diagnostic clinique de la colique néphrétique  Douleur de flanc / fosse lombaire d’installation brutale avec bandelettes urinaires et/ou imagerie correspond au diagnostic |  |  |
| EVA à la sortie < 3 |  |  |

| ***Critères d’exclusion***  ***(1 ou plusieurs critères impose la non-inclusion du patient)*** | oui | non |
| --- | --- | --- |
| Incapacité à apprécier la douleur selon l’EVA |  |  |
| Femme enceinte ou allaitante |  |  |
| Insuffisance rénale avec clairance créat < 60 ml/min/1.73m^2^ |  |  |
| Insuffisance hépatique connue |  |  |
| Allergie connue aux composants |  |  |
| Pathologie hémorragique ou usage des anticoagulants |  |  |

| ***Antécédents*** | oui | non |
| --- | --- | --- |
| Colique néphrétique |  |  |
| Lithiase rénale |  |  |
| Malformation uro-génitale |  |  |
| Infections urinaires |  |  |
| Diabète |  |  |
| Autres : ………………………………………………………………………………………………………  ………………………………………………………………………………………………………………….. |  |  |

| ***Examen clinique*** | | | |
| --- | --- | --- | --- |
| Température : °C | | | |
| EVA _0_ : | | | |
| FC _0_ : bmp | FR0 : SpO20 : | | |
| TA 0 : / mmHg | | | |
|  | ***oui*** | ***non*** | ***Siège*** |
| Sensibilité abdominale |  |  |  |
| Giardano |  |  | DT □ G □ |
|  | | |  |
| Evolution paroxystique |  |  |  |
| Irradiation aux OGE |  |  |  |
| Autres : | | | |

| ***Examens complémentaires*** | | | | | | |
| --- | --- | --- | --- | --- | --- | --- |
|  | Fait | Non fait | | Si fait | | |
|  |  |  |  | positif | | négatif |
| ECBU / Bandelette urinaire |  |  | |  | |  |
| AUSP |  |  | |  | |  |
| Créatinine (µmol /l) |  |  | |  | |  |
| Glycémie (mmol/l) |  |  | |  | |  |
| Autres : …………………………………………………………………………………….  ………………………………………………………………………………………………… |  |  | |  | |  |
| ***Echographie rénale à l’admission*** | | | | | | |
| Date : Heure : | | | | | | |
|  | | | ***oui*** | | ***non*** | |
| Calcul visible | | |  | |  | |
| Si oui, coté : DT □ G □ | | | | | | |
| Dilatation pyélo-calicielle | | |  | |  | |
| Si oui, coté : DT □ G □ | | | | | | |
| Autres anomalies rénales : | | | | | | |
| Autres diagnostics : | | | | | | |

| ***Orientation après 7 jours de sortie*** | ***oui*** | ***non*** |
| --- | --- | --- |
| **Récidive de la douleur** |  |  |
| Délai (jours): | | |
| **Reconsultation** |  |  |
| Délai (jours) : | | |
| Motif : | | |
| Echographie après sortie |  |  |
| **EVA actuelle :** | | |

| **Effets indésirables liées aux traitements** |
| --- |
| Epigastralgies : Oui □ Non □ Vomissements : Oui □ Non □ Vertiges : Oui □ Non □  Rush cutanée : Oui □ Non □ Insomnie : Oui □ Non □ Anxiété : Oui □ Non □  Diarrhée : Oui □ Non □ Somnolence : Oui □ Non □ Nausées : Oui □ Non □  Palpitations : Oui □ Non □ saignement : oui Non Démangeaisons : oui Non  BO : oui Non Vision floue : Oui Non Pâleur : Oui non  Malaise : oui Non Sudation : Oui Non |

| **Satisfaction vis-à-vis la prise en charge** | | | |
| --- | --- | --- | --- |
| **Non satisfait** | **Peu satisfait** | **Satisfait** | **Très satisfait** |
|  |  |  |  |
